# Evaluation of Malaria Diagnostic Methods as a Key for Successful Control and Elimination Programs

**DOI:** 10.3390/tropicalmed5020102

**Published:** 2020-06-19

**Authors:** Afoma Mbanefo, Nirbhay Kumar

**Affiliations:** Department of Global Health, Milken Institute School of Public Health, The George Washington University, Washington, DC 20052, USA

**Keywords:** malaria, malaria diagnosis, microscopy, RDT, PCR, malaria control, malaria elimination, resource-limited settings

## Abstract

Malaria is one of the leading causes of death worldwide. According to the World Health Organization’s (WHO’s) world malaria report for 2018, there were 228 million cases and 405,000 deaths worldwide. This paper reviews and highlights the importance of accurate, sensitive and affordable diagnostic methods in the fight against malaria. The PubMed online database was used to search for publications that examined the different diagnostic tests for malaria. Currently used diagnostic methods include microscopy, rapid diagnostic tests (RDT), and polymerase chain reaction (PCR). Upcoming methods were identified as loop-mediated isothermal amplification (LAMP), nucleic acid sequence-based amplification (NASBA), isothermal thermophilic helicase-dependent amplification (tHDA), saliva-based test for nucleic-acid amplification, saliva-based test for *Plasmodium* protein detection, urine malaria test (UMT), and transdermal hemozoin detection. RDT, despite its increasing false negative, is still the most feasible diagnostic test because it is easy to use, fast, and does not need expensive equipment. Noninvasive tests that do not require a blood sample, but use saliva or urine, are some of the recent tests under development that have the potential to aid malaria control and elimination. Emerging resistance to anti-malaria drugs and to insecticides used against vectors continues to thwart progress in controlling malaria. Therefore, future innovation will be required to enable the application of more sensitive and affordable methods in resource-limited settings.

## 1. Introduction

For centuries, humans have been plagued with malaria, a disease that seems to prevail over strategies used to combat it. It is becoming more challenging through the emergence of antimalarial drug resistance. The female *Anopheles* mosquito serves as a competent vector to transmit the *Plasmodium* parasite to human hosts with each blood meal. The mosquito remains unharmed by the parasite, and its ubiquitous nature ensures the transmissibility of the disease. According to the World Health Organization (WHO), there were 228 million cases and 405,000 deaths worldwide in 2018 [1]. The countries where the disease is endemic are referred to as the “malaria belt”. These locations mostly have tropical climates that are conducive to the breeding of mosquitoes and subsequent transmission of the parasite to human hosts. However, several countries that were in this category have managed to eliminate the disease, and have been declared “malaria free” by the WHO, due to a combination of programmatic approaches that involve early diagnosis and treatment [2]. The novel malaria RTS,S subunit vaccine seems to be a promising start for the use of vaccination as a strategy towards malaria elimination, however with a vaccine efficacy of only 30% to 50%, there is still a long way to go before vaccination can be considered a reliable method against malaria [3]. Until such time, current control measures need to be improved and deployed to their maximum capacity. 

There are four well-established species of the malaria parasite that infect humans, namely *Plasmodium falciparum, P. vivax, P. ovale*, and *P. malariae. P. falciparum*, the deadliest species, accounts for 99.7% of infections in Sub-Saharan Africa. *P. vivax* is the most common in the Americas and accounts for 75% of infections [1]. For Asia and Oceania, the number of *P. falciparum* and *P. vivax* infections are relatively equivalent. *P. ovale* and *P. malaria* are widely dispersed, but have low incidence [4]. An additional species, *P. knowlesi*, is a simian malaria parasite that is usually found in long-tailed and pig-tailed macaques. However, zoonotic human infections have been reported in Southeast Asia [5]. Malaria treatment success relies on prompt diagnosis and recommendations of the most appropriate treatment regimen. Artemisinin-based combination therapy (ACT) is recommended by the WHO for treatment of uncomplicated *P. falciparum* malaria, and ACT or chloroquine are recommended for *P. vivax, P. ovale, P. malariae* and *P. knowlesi* malaria infections. Primaquine is recommended to prevent the relapse of *P. vivax* and *P. ovale* infections. These recommendations are modified for special groups such as pregnant or lactating women, patients with other comorbidities, individuals with glucose-6-phospate dehydrogenase (G6PD) deficiency individuals and young children and infants [6]. 

Malaria is a febrile illness and clinical symptoms of uncomplicated malaria include fatigue, headaches, muscle aches, malaise, abdominal discomfort, fever, nausea and vomiting [7]. Specific diagnostic methods are needed to differentiate between malaria and other febrile illnesses. The early diagnosis of malaria can prevent further progression and lower the severity of the disease. This is especially critical for children under five years of age who accounted for about 67% of the deaths in 2018 due to severe malaria worldwide [1]. For the most effective treatment of malaria, it is important to know the species of *Plasmodium* a person is infected with and the parasitic burden in the blood. Accurate, prompt and affordable diagnostic tools are also vital for tracking successes or drawbacks of control and elimination efforts, and for future programs aimed at global malaria eradication. Active surveillance of the disease in each geographical area is essential for a program to succeed. The WHO Global Technical Strategy for Malaria aims, by 2030, to reduce malaria case incidence and mortality rates globally by 90%; to eliminate malaria from 35 countries in which malaria was transmitted in 2015; and to prevent the re-establishment of malaria in all countries that became malaria-free [8]. These targets, though ambitious, are important to set in order to challenge and remind the world that malaria is an important public health problem in need of serious and expanded efforts. 

Even with the existing challenges, several countries have succeeded in eliminating malaria. Countries such as, Algeria, Argentina, Paraguay, Sri Lanka and the Maldives have been declared malaria free by WHO in recent years [2]. This shows that with appropriate programmatic approaches, malaria incidence can be substantially reduced. The goal of eradicating malaria by 2050 set by the Lancet commission [9] is an ambitious feat and may be possible only if all stakeholders are equally committed to the goal of eradication. The Lancet commission also emphasized the importance of ultrasensitive rapid diagnostic tests (RDTs) as essential tools in the identification of asymptomatic infections and infections in pregnant women, and the need for novel diagnostic tools that do not require a finger-prick blood sample [9]. 

## 2. Materials and Methods 

**Figure 1:**
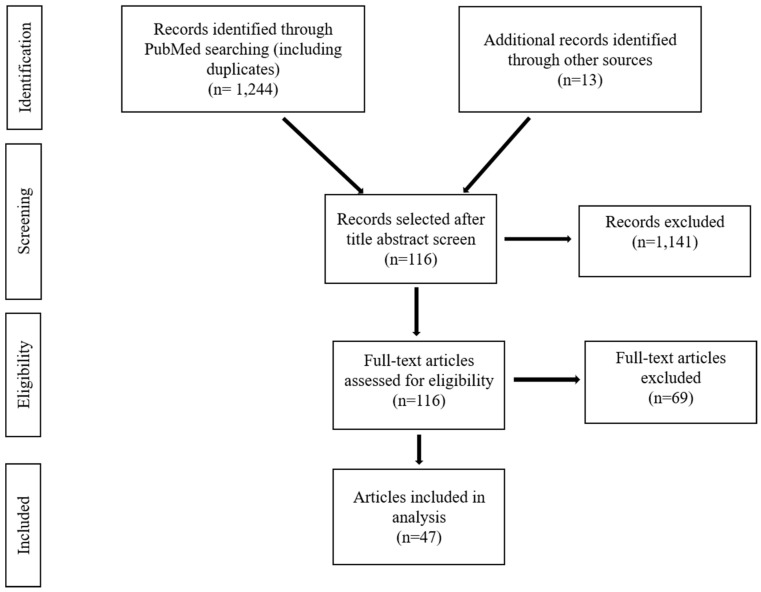
Criteria of selection of articles for review.

A systematic literature review was conducted using the PubMed online database. Review and non-review articles were identified using the following search terms, “malaria diagnosis”, “malaria control and elimination diagnostics”, “malaria diagnosis challenges”, “saliva malaria diagnostic”, “Innovative malaria tools” and “Asymptomatic malaria”. The literature search was conducted in September–October of 2019 and January 2020. It included majority review and a few non-review articles, published between 2009 and 2020, written in the English language, involved research about humans only and explained in great detail about malaria diagnostic methods or the challenges related to malaria diagnosis. The total number of articles found in the database was 1244. Thirteen additional articles were identified using Google Scholar and the WHO website. Through the careful reading of article titles and abstracts, elimination of duplicates, 47 articles were selected for full-text review (Figure 1). We provide a brief review of current and under development malaria diagnostic options and diagnostic challenges. 

## 3. Results

### 3.1. Current Malaria Diagnostic Options

#### 3.1.1. Microscopy

**Figure 2:**
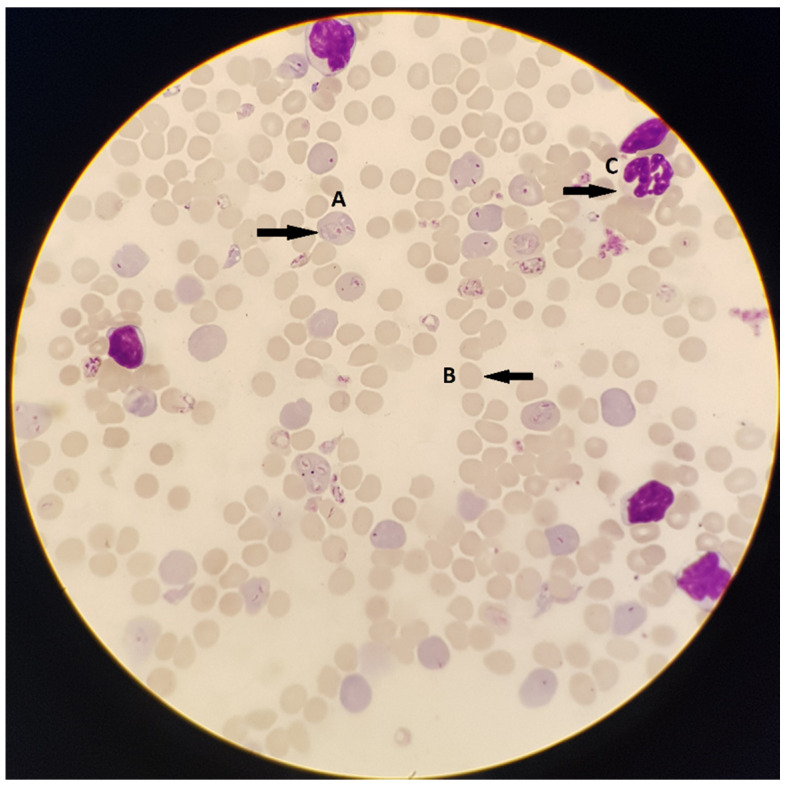
Microscopy of a thin smear *P. falciparum* infected erythrocytes stained with Giemsa. (**A**) A red blood cell infected with two malaria parasites in the “ring” stage as seen under a microscope at 100× oil immersion. (**B**) A normal uninfected red blood cell. (**C**) A normal leukocyte.

The microscopic examination of thick and thin blood films is a “gold standard” test that is used to detect parasitemia in the blood and guiding appropriate treatment. A drop of blood is collected from a patient via a finger stick or venipuncture. When a venipuncture is used for blood collection, it is suggested that the blood is spread onto a slide immediately after collection to prevent prolonged exposure to anticoagulants in the collection tube that may alter parasite morphology [10]. Thick smears are more sensitive and involve placing one to two drops of blood on a slide in a circle. The red blood cells are lysed and the various malaria parasite blood-stages, trophozoites, gametocytes and schizonts are released. Thin smears are used to detect the morphology of the parasite species and are prepared by spreading a drop of blood across a slide to create a feathered edge that contains a single layer of cells (Figure 2) [10]. The slide was stained with the Giemsa stain and examined using an Olympus bright-field microscope (BH-2, Tokyo, Japan) (100× oil immersion) by a trained laboratory personnel. The sensitivity and specificity for this method is 95% and 98%, respectively when polymerase chain reaction (PCR) is used for comparison (Table 1) [11]. The limit of detection for this method is approximately 50-200 parasites per μL of blood [12,13]. A skilled laboratory personnel is able to quantify parasitemia in a blood smear in about 60 min. Excluding the cost of labor and obtaining a microscope, each test costs approximately $0.12–$0.40 [13]. 

##### Advantages and Limitations

A blood film examination under a microscope allows for the identification of parasitemia percentage, parasitic morphology and speciation. This method requires trained personnel and sensitivity and specificity may vary based on the skill of personnel. The time it takes for parasitemia detection and quantification is long and may lead to delay in treatment. The limit of detection is also not ideal, because sub-microscopic asymptomatic individuals with low parasitemia remain undiagnosed and untreated, and also enable the transmission cycle to continue in the community.

#### 3.1.2. Rapid Diagnostic Test (RDT)

RDTs are designed to detect antigens and they involve the use of an immunochromatographic strip where the blood is dropped into one end and the results are depicted by lines on the strip surface [14]. Three types of antigens have been employed in this method, *Plasmodium* histidine-rich protein (HRP) 2 (pHRP-2), *Plasmodium* lactate dehydrogenase (pLDH) and *Plasmodium* aldolase. pHRP-2 is specific to *P. falciparum*, while pLDH and *Plasmodium* aldolase are found in all species [15]. More than 90% of commercially available RDTs target pHRP-2 [16]. Antibodies immobilized on the surface of the test strip detect these parasitic antigens when the blood and buffer mixture migrate across it. Each RDT contains a positive control to indicate the validity of the test. Currently available species-specific RDTs are only able to identify *P. falciparum* and *P. vivax* species [17]. For other species, the RDT is only able to indicate the presence of the parasite alone without speciation. 

The WHO, in collaboration with the Foundation for Innovative New Diagnostics (FIND) and the Centers for Disease Control and Prevention and other partners, conducted a review of various commercially available RDT brands. The WHO selection criteria for procurement of RDTs is used to evaluate each brand, and performance is noted with a panel detection score [18]. The WHO recommends proactive and reactive post market surveillance to ensure that each lot deployed for use is up to standard [19]. The sensitivity of RDTs ranges from 85% to 94.8% and the specificity ranges from 95.2% to 99% (Table 1) [13,20]. The limit of the detection is comparable to that of microscopy, 50–100 parasites per μL of blood, and trained personnel are able to produce results in 15 to 20 min from the time of blood collection [12]. RDTs are relatively affordable with each test costing about $0.85 [11]. An ultra-sensitive rapid diagnostic test is currently under development and could have the potential for up to 10-fold better detection limit than that of currently used RDTs, and can detect incidence of disease up to a day and a half sooner [15]. 

##### Advantages and Limitations

RDTs represent a fast and affordable method for malaria diagnosis [7]. RDTs are easy to deploy in resource-limited and hard-to-reach settings. The personnel training required is much less intensive as compared to microscopy and PCR. Community health workers are able to perform the test in their communities then prescribe treatment or refer patients to healthcare centers. False negatives are becoming more common due to parasites with pHRP-2 gene deletion and prozone phenomenon in patients with high parasitemia [14]. RDTs may be unable to keep up with the ever-evolving nature of the malaria parasite and its changing epidemiology. The RDT method does not allow for the quantification of parasitemia and consequently monitoring therapy effectiveness is difficult. It can also result in false positives because it detects pHRP-2 which can remain in the blood up to 30 days after treatment and effective elimination of an active infection [14]. The limit of detection does not allow for the identification of asymptomatic carriers, and the variation in performance of different RDT brands could lead to decreased reliability of the method.

#### 3.1.3. Polymerase Chain Reaction (PCR)

**Figure 3:**
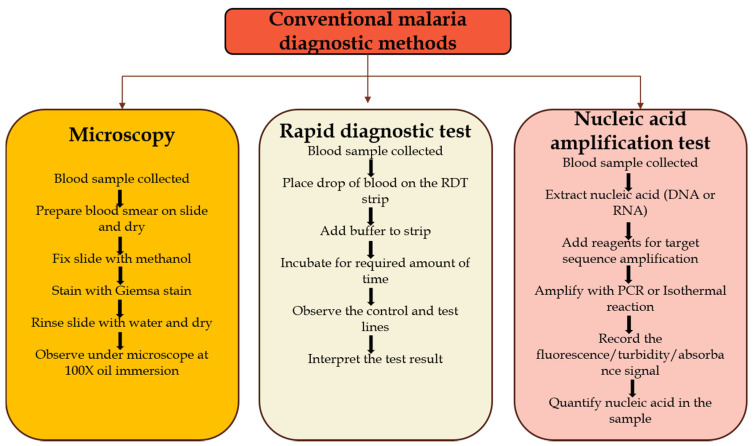
Flowchart of the currently used malaria diagnostic methods. Adapted from Ragavan, K.V. et al. [24].

PCR-based methods identify the presence of malaria target genes in a blood sample. There are various modifications of this test including, nested conventional PCR, multiplex real-time PCR and reverse transcriptase PCR [22]. Most of these methods target genes on the 18S rRNA of the malaria parasite [11]. PCR-based tests can be used for initial testing of suspected malaria cases and parasite species; however, microscopy is often used to quantify parasitemia (Figure 3). PCR-based tests are uniquely useful to identify asymptomatic and submicroscopic patients that microscopy and RDTs miss. The sensitivity and specificity for the various PCR types ranges from 98% to 100% and 88% to 94%, respectively when microscopy was used as the gold standard [23], and the limit of the detection is 0.5–5 parasites per μL of blood (Table 1) [11]. The test is typically completed in two hours, and apart from the initial cost of an expensive thermocycler and other equipment, each test costs $7 to $8 [21].

##### Advantages and Limitations

The PCR-based methods are particularly useful in parasite detection in individuals with low parasite burden. The sensitivity and specificity are higher than that of RDTs and microscopy (Table 1). PCR-based methods require the acquisition of a thermocycler which may be a financial hindrance for resource-limited settings to adopt the method. It also needs highly skilled personnel to perform the test and is not feasible for use in field settings. The PCR method also does not provide an easy method of estimating parasite burden that is often used by clinicians to make treatment decisions.

### 3.2. Novel Malaria Diagnostic Options under Development

#### 3.2.1. Loop-mediated Isothermal Amplification (LAMP)

LAMP is a relatively newer method for nucleic-acid amplification first described in 2000, and further modified for ease of visualization of amplified product using a fluorescent or colorimetric dye such as calcein and hydroxy naphthol blue (HNB) respectively [25,26]. Unlike PCR, the LAMP procedure can be carried out in a 65 °C bath or in a heat block for 30 to 60 min [21]. The sensitivity of LAMP ranges from 98.3% to 100% and specificity from 94.3% to 100% when compared to microscopy [26]. Commercially available test kits target the 18S rRNA of the malaria parasite, just like PCR [27]. LAMP performed with mitochondrial DNA targets has also been shown to have greater sensitivity and takes less time than that of 18S rRNA [27]. The limit of detection for this method is 1–5 parasites per μL of blood and the cost per test is estimated to be around $5.31 (Table 2) [21]. 

##### Advantages and Limitations

The limit of detection by LAMP is comparable to that of PCR because they are both in the range of 0.5–5 parasites per μL of blood. It is faster than PCR and the results can be assessed visually without the need for any expensive thermocycler. However, the method requires moderately skilled personnel and has a complex primer design. 

#### 3.2.2. Nucleic Acid Sequence-Based Amplification (NASBA) 

NASBA is a diagnostic method that involves the use of three enzymes, reverse transcriptase, T7 RNA polymerase and RNase H, to amplify RNA targets in a double-stranded DNA background [21]. The RNA target, such as 18S RNA, is copied into complementary DNA (cDNA) using reverse transcriptase and then the cDNA is amplified using T7 RNA polymerase [11]. It does not require the use of a thermocycler because the reaction can be carried out at 41◦C resulting in more than 10^8^-fold amplification of the target RNA sequence. The sensitivity of the method when compared to microscopy ranges from 97.40% to 100% while the specificity ranges from 80.90% to 94% (Table 2) [21]. The limit of detection is 0.01–0.1 parasites per μL of blood. The test is estimated to take about one hour to complete and costs between $5 and $20 per test [11].

##### Advantages and Limitations

NASBA, like LAMP, does not require a thermocycler and has a very low limit of detection. However, it requires extensive training of personnel to ensure the reliability of the results and the cost per test is much higher than other methods. 

#### 3.2.3. Isothermal Thermophilic Helicase-Dependent Amplification (tHDA) 

In tHDA, the double-stranded DNA is separated by helicase and single-stranded DNA-binding proteins are attached to the separated strands. Specific primers bind to the strands and DNA polymerase synthesizes new strands, and the test is performed at 65^o^C in about two hours [21]. In the application of tHDA for malaria diagnosis, the 18S rRNA gene is amplified from whole blood directly without heat denaturation or nucleic acid amplification. Probes labeled with either fluorescein (FAM) or digoxigenin (DIG) hybridize to the amplicon and the amplification product is detected with a lateral-flow strip that contains anti-FAM or anti-DIG antibodies. The sensitivity and specificity of this method are 96.6% and 100%, respectively when microscopy is used as the gold standard (Table 2) [28]. The limit of detection is 200 parasites per μL of blood and the results can be obtained in one to two hours [21,28].

##### Advantages and Limitations

The tHDA method does not require the use of a thermocycler so the cost may be more affordable than PCR. In addition to its more affordable cost, whole blood can be used directly without any manipulation, hence simplifying the method. However, the limit of detection is higher than any other nucleic acid-based methods. Although the method requires minimally trained personnel, the higher limit of detection is not suitable to detect malaria in patients with low parasitic burden. 

#### 3.2.4. Saliva-Based Test with Nucleic-Acid Amplification

This saliva-based malaria diagnosis involves the detection of a *Plasmodium* gene, 18S rRNA or P. falciparum dihydrofolate reductase gene in saliva using a nested-polymerase chain reaction (nPCR). A nPCR involves the same procedure as conventional PCR but uses two primer sets and has two successive PCR steps. The product from the first PCR reaction serves as the template for the second reaction [29]. A thermocycler is required for the procedure, and nucleic acid has to be extracted first from the saliva sample before nPCR is performed. The sensitivity and specificity of this method ranges from 86.36% to 95% (Table 2). The specificity ranges from 93% to 98.4% when compared with microscopy [29,30]. The limit of detection for this method is 1–10 parasites per μL of blood [31]. 

##### Advantages and Limitations

Saliva-based tests are noninvasive and require less training for health personnel for sample collection. However, the method still involves the use of PCR and health personnel will need advanced training on the actual PCR protocol. The procedure takes approximately six hours to complete, so this can be a major hindrance for its implementation. It can be estimated that the cost of the test will be similar to that of blood-based PCR test since it requires two PCR reactions using a thermocycler. 

#### 3.2.5. Saliva-Based Plasmodium Protein Detection

This saliva-based test detects the presence of specific *Plasmodium* proteins in the saliva of an infected person even before symptoms begin. A study conducted in Nigeria [32] used a commercially available kit, OptiMAL-IT dipstick (RDT), to detect pLDH in the saliva of children. The sensitivity of the test using whole saliva was 77.9% (Table 2) [32]. However, a study in Mali [33] reported a higher sensitivity of 97.2% and a specificity of 95.4% when compared to microscopy [33]. The limit of detection for this method is high at about 1000 parasites per μL of blood. In a separate study [34] conducted in Cameroon, Zambia and Sierra Leone, a prototype lateral-flow immunoassay (LFIA) was developed to detect the presence of PSSP17 protein, a female gametocyte protein that is presumably more abundant in saliva samples. The result is visible through the use of a handheld ultraviolet light-emitting diode flashlight. When the flashlight is used on the test strip, fluorescence is emitted and visible with the naked eye. The limit of detection for the method is ranges from 1 to 10 gametocytes per μL of blood [34]. The test can be completed between 3 and 30 min. The approximate sensitivity of the method in symptomatic patients was 83% (95% CI, 61 to 95) when compared to PCR as the gold standard [34]. 

##### Advantages and Limitations

The saliva-based diagnosis in noninvasive and the pLDH method employed is similar to that of blood-based RDTs. The sensitivity varies and test results need to be confirmed using microscopy. The limit of detection is also too high and more research needs to be carried out to improve it to an acceptable range. It has the potential to be used not only for diagnosis of symptomatic patients but also for asymptomatic carriers. This may be especially advantageous for control programs to identify and treat carriers of the parasite contributing to malaria transmission. The sensitivity of the test is comparable to that of current RDTs. Although the test is not yet commercialized, it can be estimated that the cost will be comparable to that of current blood-based RDTs. Like blood-based RDTs the test is not quantitative and will not be useful for determining parasitemia percentage in patients. 

#### 3.2.6. Urine-Based Malaria Test

Urine malaria tests involve the detection of *Plasmodium* protein pHRP-2. A commercially available test, known as the urine malaria test (UMT), involves dipping the test strip into a urine sample for two minutes, followed by incubation for twenty minutes [39]. Just like the commonly-used RDT, a positive result is indicated by dark-colored lines on the test strip. However, the differentiating factor is that this method does not involve a finger stick and can be carried out non-invasively. Multicenter clinical trials conducted in Nigeria with febrile and afebrile patients, using the UMT, have confirmed the sensitivity and specificity of the method to be 79% and 89%, respectively when compared to BinaxNOW Malaria Test kit (Table 2). The sensitivity and specificity increased to 93% and 83%, respectively when used among febrile children under five years old [35]. The limit of detection for this test is 125 parasites/µL, and each test costs about $1.50 [13,36]. 

##### Advantages and Limitations

The urine-based malaria test is relatively affordable and does not require expensive equipment or highly trained personal. The limitation for this test is that it only detects pHRP-2 from *P. falciparum* parasites. 

#### 3.2.7. Transdermal Hemozoin Detection

This method involves the detection of hemozoin-generated vapor nanobubbles using an ultrasound sensor. Hemozoin is the by-product of hemoglobin digestion by blood-stage malaria parasites. A short laser pulse administered to blood vessels through the skin localizes heat and evaporates the liquid around the hemozoin crystals. This liquid evaporation creates expanding and collapsing small sized vapor nanobubbles inside the malaria parasite [37]. After laser is activated, the probe is able to detect acoustic pulse and generates an electrical signal as an acoustic trace. A patient with confirmed malaria was tested using the prototype, and hemozoin-generated vapor nanobubbles were detected. The short laser pulse administered is skin-safe and the test is estimated to take only a few seconds to perform and is able to detect 0.00034% parasitemia (Table 2) [38]. Since hemozoin clears from the blood within nine days, compared to pHRP-2 which is cleared only after several months, this method is expected to produce few false-positive results [40]. 

##### Advantages and Limitations

The main advantages of this method are that it is noninvasive, requires no reagents and the results can be obtained within seconds. The method has a very low limit of detection and may potentially detect subclinical carriers which is helpful for disease surveillance. Highly trained personnel will be needed for initial deployment of the method in both clinical and field settings. Researchers estimated that a battery-powered device will cost about US $15,000, however the cost of a single test could be lower than that of RDTs because a single device can be used for larger number of patients [38]. Skin color has also been found to impact the results of the test and more studies are needed to address this issue. In addition, there is need for further studies to determine the safety of extended laser application, even at low pulses, to the skin. 

### 3.3. Challenges

#### 3.3.1. Malaria Diagnosis in Resource-limited Settings 

A major challenge in achieving malaria elimination in endemic settings is resource limitation. Affordable, Sensitive, Specific, User-friendly, Robust and Rapid, Equipment-free and Deliverable (ASSURED) are the criteria set by the WHO that are used to determine the suitability of a diagnostic test for use in resource-limited settings [11]. Although many malaria diagnostic methods meet one or more of the ASSURED criteria, none have met all of them. Malaria microscopy, although highly sensitive, has not met all of the ASSURED criteria due to its reliance on electricity and highly trained personnel. Sensitivity also varies depending on the laboratory technician’s experience. RDTs are currently widely used due to their non-reliance on electricity, low cost, low personnel training requirements, and rapid results. Their implementation is hindered because the limit of detection is not low enough to identify people with low parasitemia and the emergence of pHRP-2 gene deletion mutant parasites. PCR also requires reliable supply of electricity, highly trained personnel and is more expensive than RDTs and microscopy. Researchers developed a multiplex malaria sample ready (MMSR) assay that uses lyophilized PCR reagents and requires only the addition of water to reconstitute [41]. Although this assay is a promising move towards the broader use of PCR in resource-limited settings, the high cost, as much as $10 per test, and dependence on a thermocycler and DNA extraction remain challenges [41]. Clearly, novel diagnostic methods, some under development, will also need to fulfill all of the ASSURED criteria in order to be truly impactful for the goals of malaria elimination and possible eradication.

#### 3.3.2. Malaria Diagnosis among Symptomatic Carriers 

Asymptomatic malaria carriers are described as those who carry the malaria parasite in their system in the absence of fever or other typical malaria symptoms, and are not taking anti-malarial medication [42]. It is most common in many malaria endemic areas and has a prevalence that is four to five times more than symptomatic infections [43]. Research suggests that the high prevalence of asymptomatic parasitemia may be contributing to the acquisition of natural immunity that partially restricts parasite growth [42]. Most asymptomatic patients do not seek treatment because they are unaware of their status and therefore act as reservoir for gametocytes, and continue malaria transmission in the population [42]. Asymptomatic parasite carriers thus may be a major barrier to the success of malaria control, elimination or eradication efforts. The diagnosis of asymptomatic malaria is challenging because the parasitemia in these individuals is often submicroscopic and thus microscopy cannot detect parasites in a blood sample [44]. RDTs are also unable to detect these infections because its limit of detection is higher than the parasitic density of asymptomatic carriers [42]. Only molecular methods such as PCR are able to detect carriers due to their low limit of detection and high sensitivity [44].

#### 3.3.3. Malaria Diagnosis during Pregnancy 

The WHO estimates that in 2018, approximately 11 million pregnancies in moderate and high transmission sub-Saharan African countries would have been exposed to malaria [1]. Malaria during pregnancy, known as placental malaria (PM), is especially dangerous for both maternal and fetal health. Diagnosing PM is especially challenging because the malaria parasites sequester in the placenta and cannot be accurately detected by microscopy in the peripheral blood [16]. RDTs that detect pHRP-2 have shown to be useful tools for diagnosing PM. A study compared RDTs to microscopy and found that there was an 82.9% agreement between both methods [45]. This, in addition to other studies, displays evidence that RDTs, although not perfect, may be a good alternative to microscopy in resource-limited settings [16,45]. The PCR detection of PM using peripheral blood and placental blood samples revealed positivity rates that were > 20% above that of microscopy [46]. However, the limitations of PCR discussed above make it unsuitable in resource-limited settings. Studies of the association between PM diagnosed using microscopy, RDT or PCR have shown varying results [46]. A better method to diagnose PM is important in order to follow the WHO recommendations for intermittent preventive treatment in pregnancy (IPTp3). 

#### 3.3.4. Malaria Diagnosis to Reduce Under-five Mortality 

The majority of malaria deaths occur in children under the age of five. Children are more susceptible to the severe effects of the disease due to their underdeveloped immune systems and account for the majority of deaths. In household surveys conducted in 21 countries with moderate to high malaria burden, the prevalence of anemia in children under five was 61%. Mild anemia was 25% and severe anemia was 33% [1]. Severe anemia in combination with other severe symptoms account for most preventable deaths. Even though the percentage of febrile children in sub-Saharan Africa, seen in public health facilities and tested with either microscopy or RDT, has steadily increased from 38% in 2010 to 85% in 2018, not all febrile children are brought into clinics for care [1]. Owing to difficulties in prompt malaria diagnosis in select vulnerable high-risk groups such as pregnant women and infants, the WHO recommended chemoprevention in the sub-Sahel Africa regions with high seasonal malaria transmission [6]. 

#### 3.3.5. Malaria Diagnosis in Elimination Settings

The WHO certifies a country as malaria-free when it has achieved at least three consecutive years of zero indigenous cases. In addition to zero cases, the country must have a fully functional surveillance and response system that can detect and prevent re-establishment of indigenous transmission [47]. As of 2018, 27 countries have been awarded the WHO malaria-free certification [1]. Algeria and Paraguay were the recent additions to the list in 2019. The WHO has also identified 21 additional countries that have the potential to eliminate malaria by 2020 and is working with their governments to achieve this goal. It is hoped that many of these countries will achieve significant progress in reducing cases by the end of the year. Based on Algerian success, a well-trained health workforce, free healthcare, effective prevention measures, early diagnosis and treatment of all malaria cases, and a rapid response to disease outbreaks represent an approach to achieve such a goal [48]. Although it is normal to assume that not every malaria endemic country can implement all these strategies; it is still valuable to learn from successful countries that have achieved the goal. Early diagnosis and treatment of malaria cases is crucial to the success of elimination programs to halt continuous transmission of the parasite.

## 4. Conclusions and Future Goals

Although current diagnostic methods in use are not all perfect, they continue to play important roles in dealing with the current global malaria situation and to decrease the incidence of malaria. Numerous innovations continue in developing additional invasive as well as noninvasive and specific methods. Diagnostic tools are critical in ensuring that each patient receives the appropriate care. Currently-used methods such as microscopy, RDTs and PCR are not being utilized to their full capacity due to several barriers and limitations, such as cost, trained personnel, access to equipment, and unreliable electricity. In addition, not every febrile person is seen by a clinician and they usually rely on self-medication using antimalarial drugs purchased from local pharmacies or retailers without proper diagnosis and a prescription. This overuse and over-prescription of drugs is likely contributing to the emergence of anti-malarial resistant *Plasmodium* parasites. The “test and treat” strategy needs to become the norm everywhere to impede the emergence of drug resistance. RDTs, although not perfect, have the potential to result in a reduction of malaria incidence in endemic areas due to their ease of use and low cost. As disease incidence decreases, and asymptomatic infections become more prevalent, RDTs will no longer be useful in tracking submicroscopic carriers due to their limit of detection. Therefore, there will have to be a shift to implementing more sensitive and specific methods with lower limits of detection to diagnose and treat asymptomatic carriers. Interventions, such as providing malaria posts to every community in order to enable the diagnosis of febrile patients by a community health worker, have shown to reduce malaria incidence, if carried out properly. Malaria cases need to decline in order to reduce under-five and maternal mortality and achieve the United Nation’s sustainable development goal.

It will be important to build trust between communities and health workers and to ensure hospitals’ and pharmacy retailers’ adherence with recommendations based on test results. This will require a multi-sectoral approach and political will to advocate for the implementation of control and elimination strategies. Further considerations are warranted for placing RDTs in pharmacies, drug shops or malaria posts on one hand, and to educate people in seeking prompt treatment rather than alternative remedies to avoid hospital-based treatment on the other. Finally, the control of malaria continues to be challenged by the ubiquitous nature of the mosquito vector, widespread insecticide resistance, ever-threatening anti-malarial drug resistance, and the lack of an effective vaccine. Sensitive and accurate diagnosis and prompt treatment will continue to play a critical role in controlling malaria while other approaches become available to meet the challenge of malaria elimination. Other key requirements to achieve the goal of malaria free world will also rely on: (1) the recognition of trained community health workers paid fair wages as recognized members of the health system, (2) the establishment of malaria posts in endemic communities to provide access to diagnostics and treatment for those in hard-to-reach locations, (3) making RDTs available in local pharmacies and retail shops in resource limited settings, (4) integrating malaria control programs into other disease programs of individual country, and (5) political will for long-term commitment of national financial resources.

## Figures and Tables

**Table 1:** Currently used malaria diagnostic methods. For all methods except microscopy, sensitivity and specificity are compared to microscopy.

Method	Microscopy	RDT	PCR
Target	N/A	pHRP-2, LDH, Aldolase	18S rRNA
Sensitivity	95% ^1^	85% to 94.8%	98% to 100%
Specificity	98% ^1^	95.2% to 99%	88% to 94%
Limit of detection	50–200 parasites per μL of blood	50–100 parasites per μL of blood	0.5–5 parasites per μL of blood
Advantages	Identification of parasite morphologies, species and stage	Fast and easy to use	Low limit of detection making it easier to detect low parasitemia, High throughput
Limitations	Requires trained personnel and microscopes	Mutation in pHRP-2 leading to false negatives, Unable to quantify parasitemia	Needs expensive instrument and is not able to quantify parasitemia
Cost per test	$0.12–$0.40	$0.85	$7–$8
Time	60 min	15–20 min	2 h
References	[11,12,13]	[11,12,13,20]	[11,21]

^1^ Sensitivity and specificity percentages are when method is compared to polymerase chain reaction (PCR).

**Table 2:** Malaria diagnostic options under development. For all methods except the urine malaria test (UMT), sensitivity and specificity are when compared to microscopy.

Method	LAMP	NASBA	tHDA	Saliva-Based Test with Nucleic-Acid Amplification	Saliva-based Test with Plasmodium Protein Detection		UMT	Transdermal Hemozoin Detection
Target	18S rRNA, Mitochondrial DNA	18S mRNA	18S rRNA	18S rRNA, P. falciparum dihydrofolate reductase gene	pLDH	PSSP17	pHRP-2	Hemozoin
Sensitivity	98.3% to 100%	97.40–100%	96.6%	86.36–95%	77.9–97.2%	83%	79% (overall) 93% (children under 5 years) *^1^*	Unknown
Specificity	94.3% to 100%	80.90–94%	100%	93–98.46%	95.4%	Unknown	89% (overall) 83% (children under 5 years) ^1^	Unknown
Limit of detection	1–5 parasites per μL of blood	0.01–0.1 parasites/μL of blood	200 parasites per μL of blood	1–10 parasites per μL of blood	1000 parasites per μL of blood	1 to 10 gametocytes per μL of blood	125 parasites/µL of blood	Able to detect 0.00034% parasitemia
Advantages	Low limit of detection, faster reaction time than PCR, no thermocycler needed, high throughput	No thermocycler needed	Uses whole blood directly	Non-invasive sample collection	Non-invasive sample collection	Non-invasive sample collection	Non-invasive, fast and easy to use	Non-invasive, no reagents, fast, very low limit of detection
Limitations	Easily susceptible to contamination	Requires highly trained personnel, expensive	Limit of detection is higher than that of other methods	Needs expensive instrument and is not able to quantify parasitemia	Test is unable to quantify parasitemia percentage	Test is unable to quantify parasitemia percentage	Lower sensitivity and specificity than other methods	Needs highly trained personnel
Cost per test	<$1–$5.31	$5 to $20	Similar to that of LAMP	Similar to that of PCR	Unknown	Unknown	$1.50	Potentially more expensive than other methods due to the cost of instrument
Time	30–60 min	1–2 h	1–2 h	6 h	22 min	3 min to 30 min	25 min	Seconds
References	[21,26,27]	[11,21]	[21,28]	[29,30,31]	[32,33]	[34]	[13,35,36]	[37,38]

^1^ Sensitivity and specificity when compared to BinaxNow rapid diagnostic tests (RDT).
